# Inhibitory short peptides targeting EPS8/ABI1/SOS1 tri-complex suppress invasion and metastasis of ovarian cancer cells

**DOI:** 10.1186/s12885-019-6087-1

**Published:** 2019-09-05

**Authors:** Xuechen Yu, Chuan Liang, Yuanzhen Zhang, Wei Zhang, Huijun Chen

**Affiliations:** 1 Department of Gynaecology and Obstetrics, Zhongnan Hospital of Wuhan University, Wuhan, 430071 Hubei China; 2 Department of Cardiothoracic vascular surgery, Zhongnan Hospital of Wuhan University, Wuhan, 430071 Hubei China

**Keywords:** Ovarian cancer metastasis, Inhibitory short peptide, ABI1, SOS1, EPS8

## Abstract

**Background:**

We aimed to develop inhibitory short peptides that can prevent protein interactions of SOS1/EPS8/ABI1 tri-complex, a key component essential for ovarian cancer metastasis.

**Methods:**

Plasmids containing various regions of HA-tagged ABI1 were co-transfected into ovarian cancer cells with Flag-tagged SOS1 or Myc-tagged EPS8. Co-immunoprecipitation and GST-pulldown assay were used to identify the regions of ABI1 responsible for SOS1 and EPS8 binding. Inhibitory short peptides of these binding regions were synthesized and modified with HIV-TAT sequence. The blocking effects of the peptides on ABI1-SOS1 or ABI1-EPS8 interactions in vitro and in vivo were determined by GST-pulldown assay. The capability of these short peptides in inhibiting invasion and metastasis of ovarian cancer cell was tested by Matrigel invasion assay and peritoneal metastatic colonization assay.

**Results:**

The formation of endogenous SOS1/EPS8/ABI1 tri-complex was detected in the event of LPA-induced ovarian cancer cell invasion. In the tri-complex, ABI1 acted as a scaffold protein holding together SOS1 and EPS8. The SH3 and poly-proline+PxxDY regions of ABI1 were responsible for SOS1 and EPS8 binding, respectively. Inhibitory short peptides p + p-8 (ppppppppvdyedee) and SH3–3 (ekvvaiydytkdkddelsfmegaii) could block ABI1-SOS1 and ABI1-EPS8 interaction in vitro. TAT-p + p-8 peptide could disrupt ABI1-EPS8 interaction and suppress the invasion and metastasis of ovarian cancer cells in vivo.

**Conclusions:**

TAT-p + p-8 peptide could efficiently disrupt the ABI1-EPS8 interaction, tri-complex formation, and block the invasion and metastasis of ovarian cancer cells.

## Background

Ovarian cancer has the highest mortality rate among all the gynecological cancers [1]. Due to the lack of symptoms at early stages and effective screening strategies, 75% of patients with ovarian cancer usually have extensive metastasis at diagnosis [2]. In the past decades, advances in surgical techniques and chemotherapy have not effectively improved the survival of ovarian cancer patients [3]. Therefore, novel therapeutic strategies targeting the metastatic process of ovarian cancer are urgently needed.

Ovarian cancer mostly originates from malignant transformation of epithelial cells on the ovary surface. Unlike other solid tumors that rely on the vasculature for metastasis, ovarian cancer mainly disseminates throughout the peritoneal cavity with the flow of ascites, and implants onto the peritoneal organs [4–6]. Lysophosphatic acid (LPA) is a growth factor-like phospholipid, which can be produced and secreted into peritoneal cavity by ovarian cancer cells [6–9]. High concentrations of LPA have been found within the ascites of ovarian cancer patients [10].

LPA and its receptors have been recognized to play a critical role in the metastasis of ovarian cancer [11, 12]. Our previous study demonstrated that LPA could stimulate Rac activation, cytoskeleton reorganization, and ovarian cancer cell migration through a signaling pathway consisting of Ras-SOS1/EPS8/ABI1 tri-complex [14]. The integrity of SOS1/EPS8/ABI1 tri-complex may determine ovarian cancer metastatic potentials, as silencing any member of SOS1/EPS8/ABI1 tri-complex is sufficient to diminish the migration and metastatic colonization of ovarian cancer cells [14]. These results implicate SOS1/EPS8/ABI1 tri-complex as an ideal therapeutic target, and disrupting the tri-complex may suppress the metastatic process of ovarian cancer. Among the tri-complex, SOS1 functions as a Rac-specific guanine nucleotide exchange factor (GEF), which finally induces Rac-regulated cytoskeleton recombination and cell migration [15–17]. EPS8 acts as a substrate for the tyrosine kinase receptors. ABI1 is a scaffold protein that connects SOS1 and EPS8 [18–24]. ABI1 binds to SOS1 with its SH3 domain [25], and then to the SH3 domain of EPS8 [26]. In this study, we found that the SH3 and poly-proline+PxxDY regions of ABI1 are responsible for its interaction with SOS1 and EPS8, respectively.

The development of biology is helpful in finding therapeutic targets for diseases [27]. Biomedicine has made great progress in cancer treatment [28]. Recent studies have shown the success of using inhibitory peptides to disrupt specific protein-protein interactions and their pertinent biological events [29–32]. As small molecules, inhibitory peptides also hold tremendous promise for clinical applications. Peptides have made great progress in the fields of vaccines, antibiotics, anti-tumor drugs, and diagnostic agents [33]. Recently, short peptides have been successfully used in interfering with signaling pathways as new therapeutic tools for cancer treatment [34, 35].

In this study, we developed peptides that can inhibit the EPS8-ABI1 and ABI1-SOS1 interactions and tested their efficacies in suppressing ovarian cancer metastasis. Because of the nature of ABI1 as an adaptor protein [18, 22] and the smallest size among the three members of tri-complex, we chose ABI1 as a target for designing short inhibitory peptides. We identified the respective amino acid regions in ABI1 essential for SOS1 or EPS8 bindings, generated overlapping peptides covering these regions, and identified the peptides that could prevent the formation of SOS1/EPS8/ABI1 tri-complex. We believe that the inhibitory peptides developed in this study can represent a new line of therapeutic agents against ovarian cancer metastasis.

## Methods

### Cells and antibodies

The human ovarian cancer cell lines SK-OV3, HEY, and OVCAR3 were gifted to us by the Department of Biochemistry and Molecular Biology, Georgia Regents University (Georgia, USA). Cells were cultured in Dulbecco’s modified Eagle’s medium (DMEM) supplemented with 10% (w/v) fetal bovine serum (FBS) at 37 °C in a humidified incubator containing 5% CO_2_. LPA was purchased from Avanti Lipid (Alabaster, AL). DMEM, serum, and other cell culture supplies were from Hyclone (Waltham, MA). SOS1 mAb was purchased from Santa Cruz Biotechnology Inc. (Cat#: sc-55,528, titer: 1:2000), EPS8 mAb from BD Biosciences (Cat#: 610144, titer: 1:1000), and ABI1 mAb from MBL international Corporation (Cat#: D147–3, titer: 1:1000).

### Construction and infection of plasmids

Vectors containing the coding sequences of human EPS8, SOS1, and ABI1 were purchased from Openbiosystems (Lafayette, CO). Lentiviral vectors encoding EPS8, SOS1, and ABI1 were prepared by subcloning these coding sequence into pCDH-CMV-MCSEF1-Puro vector, and tagged with Myc, Flag, and HA, respectively. OVCAR3 cells were transfected with two of the three expression plasmids (Myc-EPS8, HA-ABI1, and Flag-SOS1) and chosen by puromycin. The efficiency of Myc-EPS8, HA-ABI1 and Flag-SOS1 overexpression was analyzed by western blotting. After 12 h starvation, cells were stimulated with 20 μM LPA and followed by Co-IP assay. To determine the regions of ABI1 responsible for EPS8 and SOS1 binding, we divided ABI1 into the following regions: WAB (aa: 1–79), SNARE (aa: 45–107), HHR (aa: 93–169), proline-rich (aa: 170–340), poly-proline (aa: 341–418), and SH3 (aa: 419–508) (Fig. 2a). We generated plasmids containing HA-tagged regions of ABI1 described above, using the pCDH-CMV-MCSEF1-Puro vector. OVCAR3 cells were co-transfected with these plasmids along with Flag-tagged SOS1 or Myc-tagged EPS8. Recombinant plasmids with Flag-tagged proline-rich region of SOS1 (aa: 1131–1333) and Myc-tagged SH3 region of EPS8 (aa: 535–586) were also prepared using the same procedure.

### Matrigel invasion assay

The Matrigel invasion assay was performed as per manufacturer’s instructions (Corning Incorporated, MA, USA). Briefly, LPA was dissolved in serum-free medium (20 μM) and added into the lower chambers of the invasion plates to induce cell invasion. Serum-free medium without LPA was used as control. Serum-starved ovarian cancer cells (10^5^/well, in log phase) were added into the chambers and allowed to invade for 48 h. The cells that remained in the chambers were removed and the invaded cells on the lower surface of the chambers were fixed and stained with crystal violet. The crystal violet-stained cells were solubilized with 10% acetic acid and quantified on a microplate reader at 600 nm. Fold increase in cell invasion was calculated to evaluate cells’ responsiveness to LPA (OD_600_ LPA-induced cell invasion/OD_600_ base cell invasion) [36].

### Co-immunoprecipitation (co-IP)

The cells were detached and resuspended in 1 ml lysis buffer. The cell lysates were centrifuged for 5 min (8000 rpm 4 °C). The supernatant was transferred into another tube and incubated with the anti-ABI1 antibody (2 μg) at 4 °C overnight. Then, γ-bound beads (50 μl) were added, and the mixture was incubated for another hour. The mixture was centrifuged for 5 min (8000 rpm 4 °C), the supernatant was carefully removed, and the beads were washed four times for 10 min. The interactions between ABI1 and the EPS8 or SOS1 were detected by western blotting using anti-EPS8 or anti-SOS1 antibody. The same procedure was adopted in all the other Co-IP experiments. ImageJ 1.41 software (National Institute of Health, Bethesda, MD, USA) was used to quantified the intensity of the western blotting bands. The expression of same protein in the cell lysis was used as an internal control. The band intensity of each sample were normalized by the cell lysis that detected the same protein, and then compared with control. Three repeats were set up for each experiment.

### GST (glutathione-S-transferase)-fusion protein pull-down assay

The BL21 competent cells were transfected with the vector expressing each domain of ABI1, incubated until they reached an OD_600_ of 0.4–0.5, and then induced by IPTG. Expressed proteins were extracted from BL21 cells and incubated with the GST Beads overnight for GST fusion proteins. The expression and purification effects were analyzed by SDS-PAGE. The lysates from ovarian cancer cells were incubated with ABI1-GST Beads for 4 h and the EPS8 or SOS1 expression was analyzed by western blotting using anti-EPS8 or anti-SOS1 antibodies. ImageJ 1.41 software was used to quantified the intensity of the western blotting bands. The same procedure was adopted in all the other GST-fusion protein pull-down assays.

### Peritoneal metastatic colonization assay

Ovarian cancer cells in log-phase were trypsinized, washed twice with PBS, and resuspended. Six-week-old athymic female homozygous nu/nu mice (Beijing Hua Fukang biological Polytron Technologies Inc.) were intraperitoneally injected with ovarian cancer cells (10^7^ cells/0.2 ml PBS/mice). Seventy-two hours after injection, mice were divided into several groups (six mice/group) and PBS, TAT-fused scramble peptides or TAT-fused inhibitory short peptides (TAT-p + p-8, TAT-SH3–3) were administered by intraperitoneal injections, at a dose of 0.5, 3 and 15 nmol/g body weight. Then, the peptides were given to the animals every 2 days for a total of 4 weeks. After which, the mice were sacrificed by cervical dislocation, autopsied. Metastatic implants were collected and weighed [37]. All animal experiment procedures were approved by the Animal Center of Wuhan University. All the procedures were performed in accordance with the relevant guidelines and regulations.

### Statistical analysis

SPSS24.0 was used for all statistical analyses. Statistical analyses were performed with ANOVA and independent *t-*test. Chi-square test and Fisher’s exact test were used to analyze the data. All the statistical tests were two-sided and a *P*-value of less than 0.05 was considered statistically significant.

## Results

### The formation of endogenous SOS1/EPS8/ABI1 tri-complex in ovarian cancer cell invasion

**Fig. 1:**
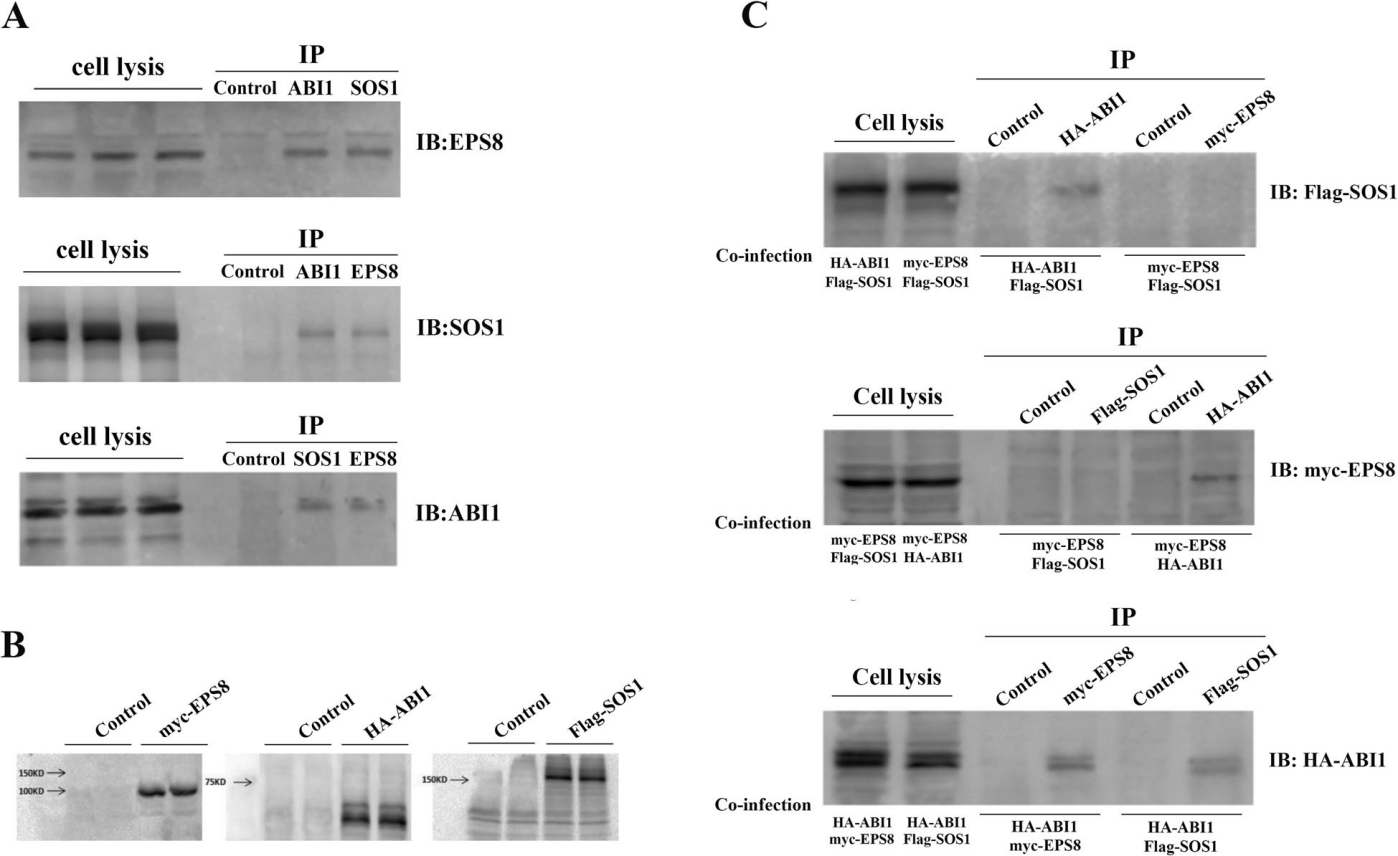
The formation of endogenous SOS1/EPS8/ABI1 tri-complex in ovarian cancer cell invasion. ABI1 served as a scaffold protein in SOS1/EPS8/ABI1 tri-complex. **a.** The metastatic ovarian cancer cell line SK-OV3 was treated with LPA for 5 min to induce cell invasion. Co-IP assay was employed to investigate the formation of endogenous SOS1/EPS8/ABI1 tri-complex after LPA stimulation. **b.** The Myc-tagged EPS8, HA-tagged ABI1 and Flag-tagged SOS1 recombinant plasmids were constructed. **c.** The OVCAR3 cells, which has no ABI1 expression, were infected by every two of the three expression plasmids (Myc-EPS8, HA-ABI1 and Flag-SOS1). Cell lysates were collected after LPA stimulation. The Co-IP assay was used to detect the interactions of ABI1-SOS1, ABI1-EPS8, and SOS1-EPS8

In vitro formation of SOS1/EPS8/ABI1 tri-complex with GST recombinant proteins have been previously reported [18, 20, 22]. We thus employed the Co-IP assay as a confirmative approach. We first treated metastatic ovarian cancer cell line SK-OV3 with LPA for 5 min to induce cell invasion. Cells were then lysed and incubated with anti-ABI1 antibody as well as γ-bound beads. Subsequently, the expression of SOS1 or EPS8 was detected by immunoblotting with respective antibodies. It was found that, in the anti-ABI1 immunoprecipitates, the other two proteins could be detected (Fig. 1a). These results demonstrated the formation of endogenous SOS1/EPS8/ABI1 tri-complex in the event of LPA-induced ovarian cancer cell invasion.

### ABI1 serves as a scaffold protein in SOS1/EPS8/ABI1 tri-complex

To determine whether ABI1 mediates the interaction between EPS8 and SOS1, as expected in the tri-complex model, the experiments have to be performed under conditions in which the endogenous formation of SOS1/EPS8/ABI1 tri-complex is disrupted. Thus, we performed Co-IP in OVCAR3 cell line, which demonstrated a lack of ABI1 expression in our previous study. We constructed Myc-tagged EPS8, HA-tagged ABI1, and Flag-tagged SOS1 recombinant plasmids (Fig. 1b). OVCAR3 cells were transfected with two of the three expression plasmids (Myc-EPS8, HA-ABI1, and Flag-SOS1). Cell lysates were collected after LPA stimulation. The results of Co-IP showed that there were interactions between ABI1-SOS1 and ABI1-EPS8, but SOS1 and EPS8 could not bind directly with each other (Fig. 1c). These results indicated the role of ABI1 as a scaffold protein in connecting SOS1 and EPS8.

### Characterizing the regions of ABI1 that mediate its interaction with SOS1 and EPS8

**Fig. 2:**
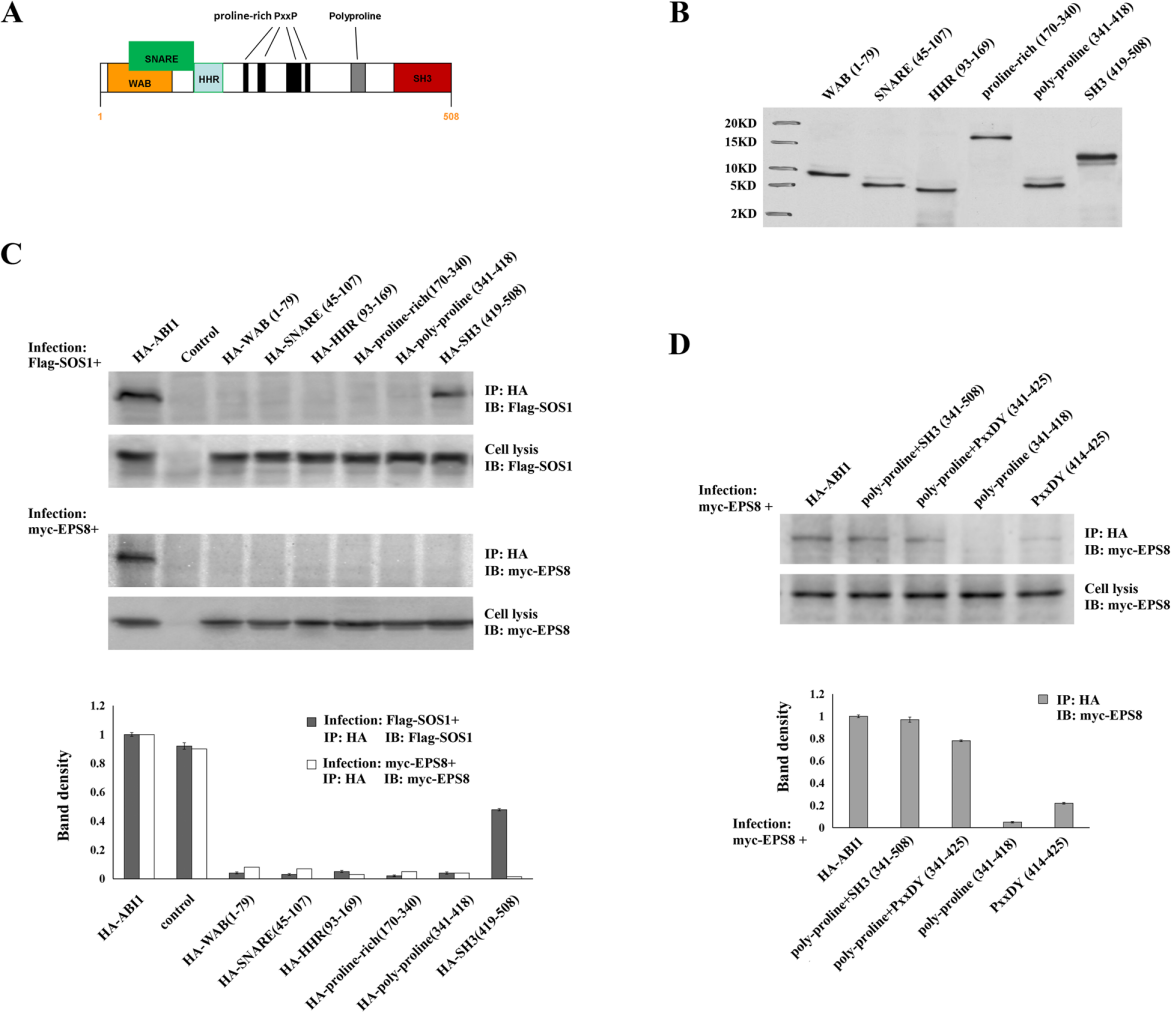
Characterizing regions in ABI1 mediating its interaction with SOS1 and EPS8. **a.** ABI1 was divided into the following regions: WAB (aa:1–79), SNARE (aa:54–108), HHR (aa:108–153), proline-rich (aa:153–331), poly-proline (aa:331–384) and SH3(aa:384–508). **b.** The plasmids containing HA-tagged various regions of ABI1 above were generated, using the pCDH-CMV-MCSEF1-Puro vector. The empty vector was used as control. **c.** The plasmids with different regions of ABI1 were co-infected into OVCAR3 cells with Flag-tagged SOS1 or Myc-tagged EPS8, respectively. Cell lysates were collected after LPA stimulation. Co-IP was performed to map out the regions in ABI1 responsible for ABI1-SOS1 or ABI1-EPS8 interactions. The results were quantified by ImageJ. The band density of each sample were normalized by cell lysis that detected the same protein and compared with control. **d.** We re-divided the C-terminal of ABI1 into the following regions: poly-proline+SH3 region (aa:331–508), poly-proline+PxxDY (aa:331–425)、poly-proline (aa:331–384)、PxxDY (aa:414–425). HA-tagged plasmids containing those regions were generated. Co-IP were performed to further identify which region mediated the ABI1-EPS8 interaction

Because ABI1 functions as a scaffold protein and has the smallest size in the tri-complex (508 aa vs. 1333 aa for SOS1 and 822 aa for EPS8), we focus on finding the regions of ABI1 that mediate SOS1 and EPS8 binding. Based on web-based protein domain searching programs and published literatures [38–45], we identified multiple motifs that could potentially mediate protein-protein interactions, and divided ABI1 into the following regions: WAB (aa: 1–79), SNARE (aa: 45–107), HHR (aa: 93–169), proline-rich (aa: 170–340), poly-proline (aa: 341–418), and SH3 (aa: 419–508) (Fig. 2a). Then we generated plasmids containing HA-tagged regions of ABI1 described above, using the pCDH-CMV-MCSEF1-Puro vector. (Fig. 2b). The empty vector was used as control. These plasmids were co-transfected into OVCAR3 cells along with Flag-tagged SOS1 or Myc-tagged EPS8. Cell lysates were collected after LPA stimulation. Co-IP was performed to map out the regions in ABI1 responsible for ABI1-SOS1 and ABI1-EPS8 interactions. The results showed that Flag-SOS1 could only be detected in the Co-IP products of anti-HA-SH3 antibody, which indicated that the SH3 region of ABI1 mediated the ABI1-SOS1 interaction (Fig. 2c). On the contrary, no expression of Myc-EPS8 was found in the Co-IP products of any ABI1 regions (Fig. 2c). Since the non-traditional proline rich domain, namely PxxDY motif, was reported to interact with SH3 domain, we re-divided the C-terminal of ABI1 into the following regions: poly-proline+SH3 (aa: 341–508), poly-proline+PxxDY (aa: 341–425), poly-proline (aa: 341–418), and PxxDY (aa: 414–425). HA-tagged plasmids containing those regions were generated. Co-IP was performed to further determine which region mediated the ABI1-EPS8 interaction. It was found that poly-proline+PxxDY region of ABI1 was responsible for its interaction with EPS8 (Fig. 2d).

**Fig. 3:**
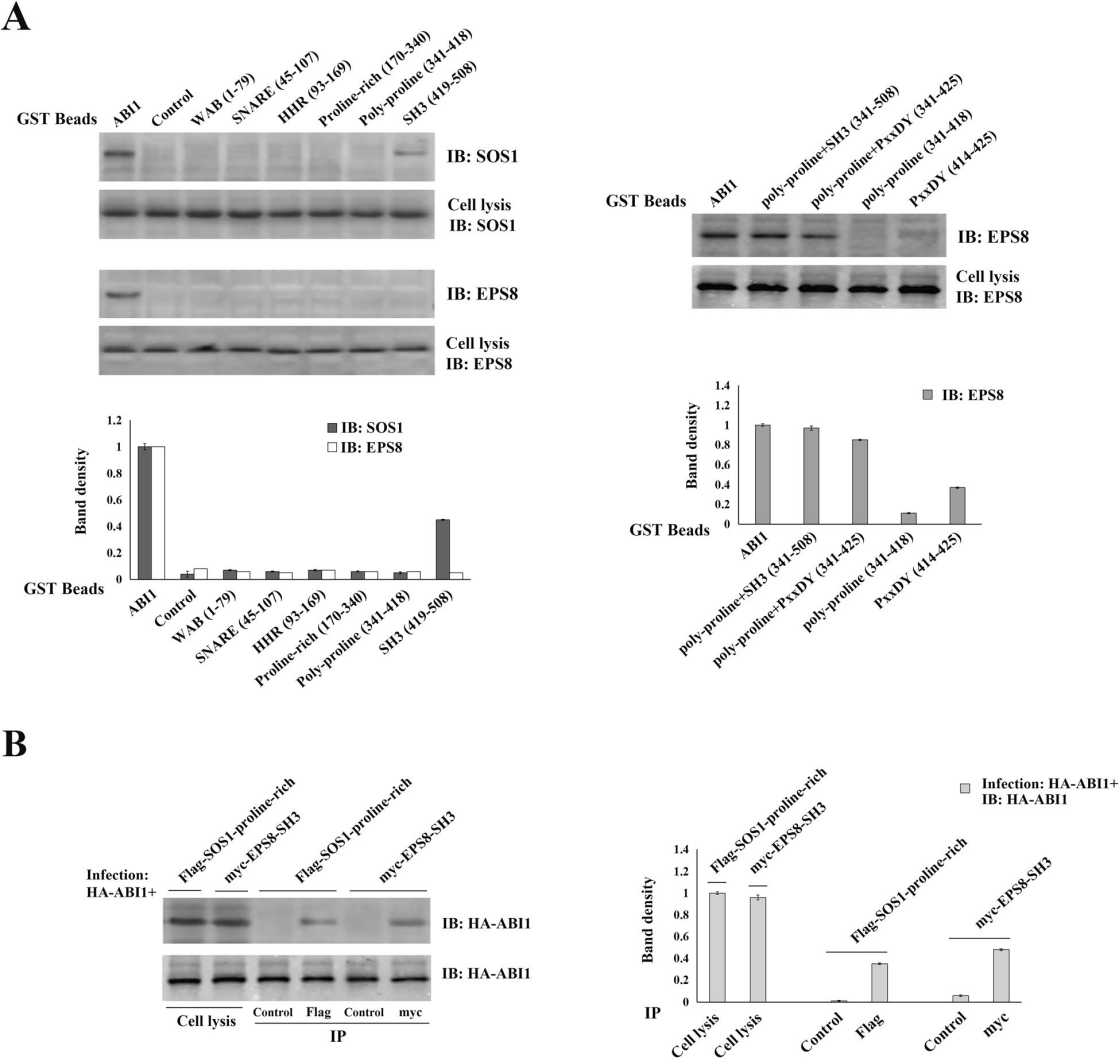
Confirming the regions of ABI1 responsible for EPS8 or SOS1 binding. Characterizing regions in SOS1 and EPS8 mediating their interaction with ABI1. **a.** GST-pulldown assay was performed as follows. Recombinant GST-fused beads with various ABI1 fragments were incubated with LPA-treated OVCAR3 cell lysates and subsequently analyzed by immunoblotting to detect SOS1 or EPS8 expression. Results from above experiments also identified the SH3 and poly-proline+PxxDY as the regions in ABI1 responsible for SOS1 and EPS8 interaction, respectively. **b.** Flag-tagged proline-rich region of SOS1 (aa:1131–1333) and Myc-tagged SH3 region of EPS8 (aa:535–586) recombinant plasmids were generated, and then co-infected with HA-tagged ABI1 into OVCAR3 cells, respectively. Cell lysates were collected after LPA stimulation. The Co-IP assay was performed to determine the regions in SOS1 and EPS8 mediating their interactions with ABI1

To verify the above results, we employed the GST-pulldown assay as a validation method. We first prepared recombinant GST-fused beads with various ABI1 fragments. These beads were incubated with LPA-treated OVCAR3 cell lysates for 2 h, and subsequently analyzed by immunoblotting to detect SOS1 or EPS8 expression. Results from these experiments also identified the SH3 and poly-proline+PxxDY as the regions in ABI1 responsible for SOS1 and EPS8 interaction, respectively (Fig. 3a).

### Characterizing the regions which mediate the interaction of SOS1 and EPS8 with ABI1

Previous studies have shown that ABI1 binds to the proline-rich domain of SOS1 through its SH3 domain, and the SH3 domain of EPS8 through its poly-proline+PxxDY region [20–22, 43]. To confirm this, we constructed recombinant plasmids with Flag-tagged proline-rich region of SOS1 (aa: 1131–1333) and Myc-tagged SH3 region of EPS8 (aa: 535–586). These plasmids were then co-transfected with HA-tagged ABI1 into OVCAR3 cells. Cell lysates were collected after LPA stimulation. The results from Co-IP demonstrated that the proline-rich region of SOS1 mediated its interaction with ABI1, while EPS8 bond to ABI1 by its SH3 region (Fig. 3-B).

### Developing inhibitory short peptides that can disrupt SOS1/EPS8/ABI1 tri-complex

Inhibitory peptides have shown great success in preventing protein-protein interactions. These peptides are generated based on the amino acid sequences essential for the respective protein interactions, and therefore have high specificity [31].

**Fig. 4:**
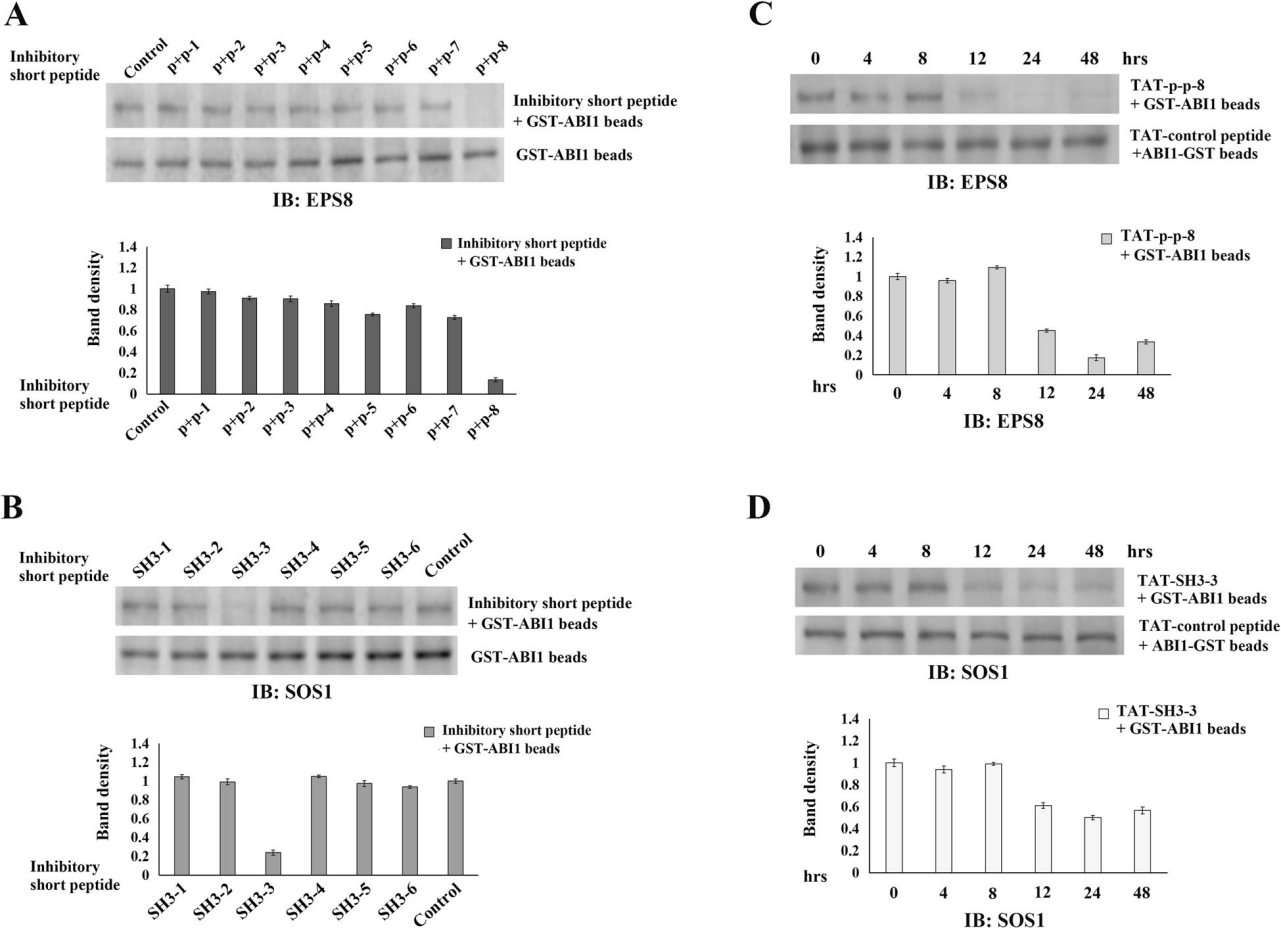
Investigating inhibitory short peptides that can disrupt SOS1/EPS8/ABI1 tri-complex. Confirming the inhibitory capability of short peptides in vivo. **a***.* we generated a series of synthetic inhibitory short peptides (each with 15 aa and overlapped by 5 aa) according to the poly-proline+PxxDY region of ABI1. After LPA stimulation, inhibitory short peptides were added to SK-OV3 cell lysates for 1 h prior to incubation with GST-ABI1 beads. GST-pulldown was employed to determine the effects of these peptides on ABI1-EPS81 binding. **b**. A series of synthetic inhibitory short peptides was generated (each is 25-amino acids long and overlapped by 5-amono acids) according to the SH3 region of ABI1. GST-pulldown was employed to determine the effects of these peptides on ABI1-SOS1 binding. **c**. The TAT-containing inhibitory short peptides or control peptides (10 μM) were added into the cell culture of SK-OV3 and incubate for different times. Then, GST-pulldown assay was performed to test the effects of TAT-containing inhibitory short peptides on ABI1-EPS8 interaction. **d**. The same experiments as above were performed to test the effects of TAT-containing inhibitory short peptides on ABI1-SOS1 interaction

In the experiments described above, we determined the regions in ABI1 responsible for binding SOS1 or EPS8. We then generated a series of synthetic inhibitory short peptides (each with 15 aa and overlapped by 5 aa) according to the sequence of poly-proline+PxxDY and SH3 regions of ABI1, through a commercial source. Efficient inhibitory short peptides are supposed to competitively inhibit the protein-protein interactions, and eventually block the formation of SOS1/EPS8/ABI1 tri-complex. GST-pulldown was employed to determine the inhibitory activity of these peptides on ABI1-SOS1 or ABI1-EPS8 interactions. Briefly, after LPA stimulation, inhibitory short peptides (10 μM) were added into SK-OV3 cell lysates 1 h prior to incubation with GST-ABI1 beads. Then the beads were washed and analyzed by immunoblotting to detect SOS1 or EPS8 expression. Unrelated scrambled peptides were used as control. Results from these experiments identified p + p-8 (ppppppppvdyedee) as the short peptide capable of blocking in vitro ABI1-EPS8 interaction (Fig. 4-a). However, none of these peptides could inhibit the interaction between ABI1 and SOS1 (data not shown).

Considering the possible effects that tertiary structure might have on protein interactions, we thought that the 15 aa long peptides may lack the tertiary structure necessary for binding. Thus, we redesigned the peptides with longer sequences. Six inhibitory short peptides were synthesized according to the SH3 (aa: 419–508) region of ABI1 (each had a length of 25 aa and overlapped by 10 aa). The result of GST-fusion protein pull-down assay demonstrated that SH3–3 (ekvvaiydytkdkddelsfmegaii) was the short peptide that could effectively inhibit the combination of ABI1 and SOS1. (Fig. 4b).

### Confirming the SOS1/EPS8/ABI1 tri-complex disrupting capability of the inhibitory short peptides in vivo

After identifying the most efficient inhibitory peptides, we further investigated their capability of preventing formation of SOS1/EPS8/ABI1 tri-complex in vivo. HIV-TAT sequence (YGKKRRQRRPP) is a very effective cell membrane-penetrating peptide, which has been widely used to transduce peptides/proteins into living cells [45]. We used this HIV-TAT sequence to modify the peptides (TAT-p + p-8 and TAT-SH3–3) for delivering them into ovarian cancer cells. The TAT-containing inhibitory short peptides were produced through commercial sources. An unrelated TAT-peptide was used as control. HIV-TAT modified inhibitory or control peptides (10 μM) were added into the cell culture of SK-OV3 and incubated for various time periods. A FITC (fluorescein isothiocyanate)-conjugated HIV-TAT peptide was also synthesized and used to test the delivery efficiency for the proposed experiments beforehand. Since the accuracy of the experiments might be affected by cell proliferation, MTT (3-(4,5-dimethyl-2-thiazolyl)-2,5-diphenyl-2-H-tetrazolium bromide) assay were also performed. By comparing the growth/proliferation index with control peptide-treated cells, we confirmed that disrupting the SOS1/EPS8/ABI1 tri-complex had no significant effect on cell proliferation (data not shown). GST-pulldown assay was then performed to test the effects of TAT-containing inhibitory short peptides on ABI1-SOS1 and ABI1-EPS8 interactions. The results showed that peptide TAT-p + p-8, which was capable of disrupting ABI1-EPS8 interaction in vitro, also efficiently blocked the binding of ABI1-EPS8 in vivo. The maximum effect was achieved at 24 h after administration of this peptide (Fig. 4c). In contrast, the peptide TAT-SH3–3 showed moderate inhibitory effect on the interaction between ABI1 and SOS1 in vivo. This might be due to the longer length and weaker penetration capability of the peptide (Fig. 4d).

### Evaluation of the efficacy of SOS1/EPS8/ABI1 targeting inhibitory short peptides in suppressing ovarian cancer metastasis

**Fig. 5:**
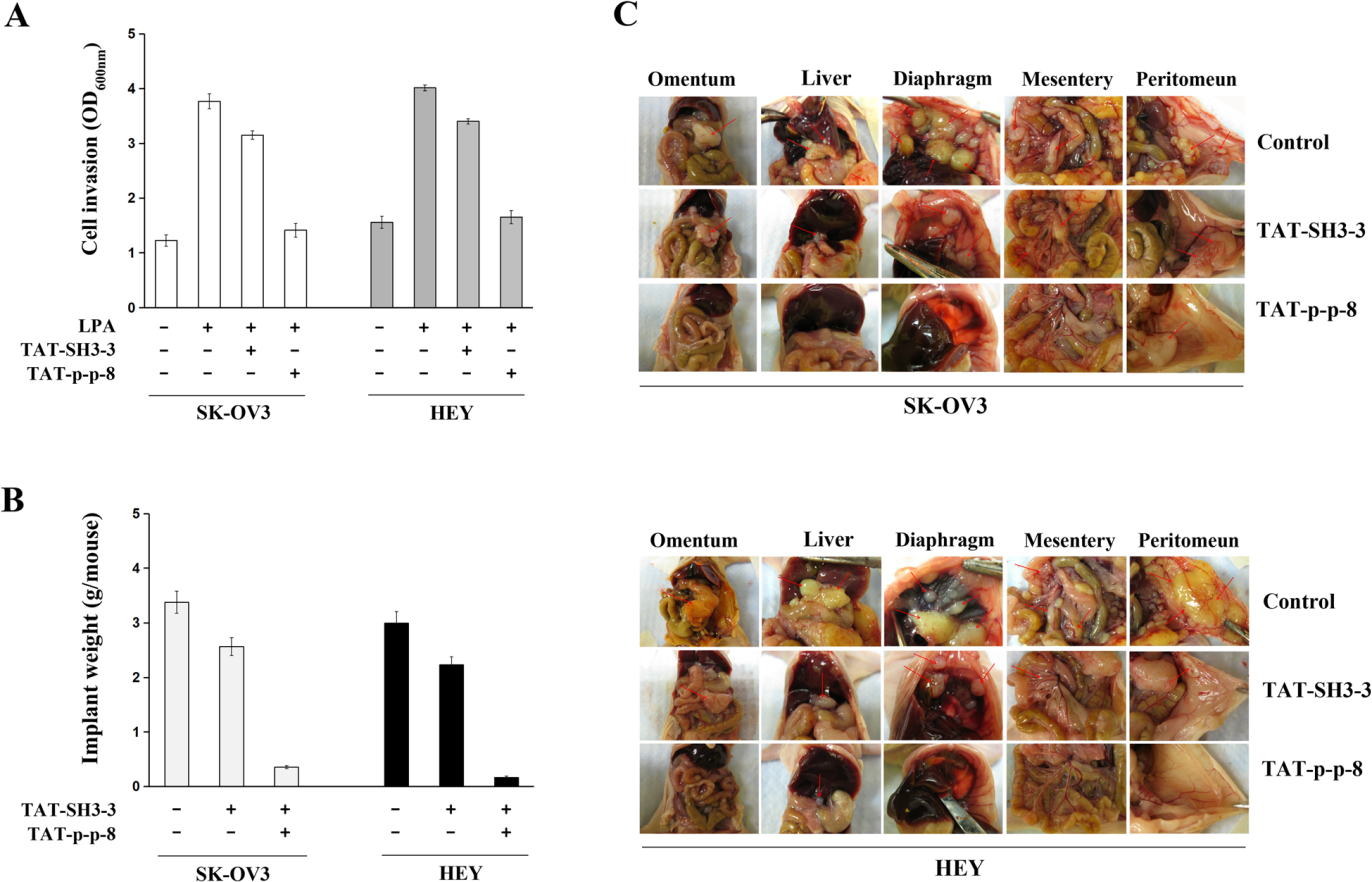
Effects of inhibitory short peptides on the invasion and metastatic colonization of ovarian cancer cells. A. SK-OV3 and HEY cells were treated by TAT-fused peptides (TAT-p + p-8 or TAT-SH3–2) for 24 h before LPA-stimulation. Matrigel invasion assay was performed to test the suppressive capabilities of these inhibitory short peptides on cell invasion. Effect of inhibitory peptides on peritoneal metastatic colonization of ovarian cancer cells. B and C. SK-OV3 or HEY cells were intraperitoneally injected into nu/nu mice. Seventy-two hrs after injection, mice were intraperitoneally injected with PBS, TAT-fused scramble peptides or TAT-p + p-8 every 2 days for four weeks, respectively. By comparing the weight of metastatic implants from animals receiving inhibitory short peptides to those receiving PBS or scrambled control peptides, we estimated the capability of inhibitory short peptides in suppressing the metastasis of ovarian cancer cells

Since the integrity of SOS1/EPS8/ABI1 is essential for LPA-induced ovarian cancer metastasis [4], these inhibitory short peptides are expected to inhibit the invasion of cancer cells. Two metastatic cell lines SK-OV3 and HEY were used in the study. The cells were treated with the TAT-fused peptides (TAT-p + p-8 and TAT-SH3–3) for 24 h before LPA-stimulation. Matrigel invasion assay was performed to test the suppressive capabilities of these inhibitory short peptides on cell invasion. It was found that the peptide TAT-p + p-8 could significantly block LPA-induced invasion in both SK-OV3 and HEY cells. These results suggested that the peptide TAT-p + p-8, which was capable of disrupting SOS1/EPS8/ABI1 tri-complex, could effectively suppress the invasion of ovarian cancer cells. The other peptide TAT-SH3–3, also showed some inhibitory effects on cell invasion, but the inhibition was not statistically significant (Fig. 5a).

We have previously used a well-established peritoneal seeding model to study ovarian cancer metastasis [13, 14]. We used this model again to test the efficacy of the inhibitory peptides in suppressing metastatic colonization of ovarian cancer cells. Briefly, metastatic ovarian cancer cell lines SK-OV3 or HEY were intraperitoneally injected into nu/nu mice. Seventy-two hours after injection, mice were intraperitoneally injected with PBS, TAT-fused scramble peptides or TAT-fused inhibitory short peptides, once every 2 days for 4 weeks. The mice were then sacrificed to collect the metastatic implants. By comparing the weight of the metastatic implants from animals receiving inhibitory short peptides to those receiving PBS or scrambled control peptides, we found that TAT-p + p-8, a TAT-fused inhibitory peptide, could significantly suppress the metastasis of ovarian cancer cells (Fig. 5b, c). The inhibitory effects of peptide TAT-SH3–3 on metastatic colonization was not as statistically significant as TAT-p + p-8. These results were in accordance with our findings of the invasion assay.

## Discussion

One of the most characteristic metastasis-promoting functions of LPA is its ability to stimulate cell migration [46]. Our previous studies have elucidated the signaling events associated with LPA-stimulated ovarian cancer metastasis [13, 14], and found that LPA induced cytoskeleton reorganization as well as cancer cell migration through Rac activation. We also identified SOS1/EPS8/ABI1 tri-complex, a Rac-GEF, as a component essential for the elevated Rac activity [14]. With the aid of a well-established peritoneal seeding model [37], we demonstrated that the presence of SOS1/EPS8/ABI1 tri-complex correlated well to the metastatic potential of ovarian cancer cells, and an intact SOS1/EPS8/ABI1 tri-complex is required for Rac activation. Therefore, we thought that investigating the interaction models of these three proteins might be helpful in developing new anticancer drugs.

SOS1 protein (150 kDa) is composed of several domains and functions as a dual GEF of Ras and Rac in different steps of signaling cascade. The C-terminal segment of SOS1 contains a proline-rich domain (PxxP), through which it interacts with the SH3 domains of ABI1. In its central segment, SOS1 has two domains, REM (Ras exchanger motif) and CDC25 (cell division cycle 25), that can catalyze the exchange of GDP-GTP in Ras. The N-terminal segment contains a Dbl homology (DH) domain in tandem with a pleckstrin homology (PH) domain, which stimulates GTP/GDP exchange for Rac. SOS1 displays Ras specificity, when associated with Grb2. On the contrary, SOS1 acts as a Rac-GEF, when engaged in a complex with EPS8 and ABI1 [43]. Grb2 and ABI1 bind to the same site on SOS1 through their respective SH3 domains, thus determining the formation of either a SOS1/Grb2 or a SOS1/ABI1/Eps8 complex, endowed with Ras or Rac-specific GEF activities, respectively [17]. EPS8, a substrate of the EGFR kinase, contains three major domains: a N-terminal phosphotyrosine binding (PTB) domain, a central SH3 domain, and a C-terminal “effector region” domain [21]. The SH3 domain of EPS8 tends to combine with the PxxDY motif, which happen to be present in ABI1, rather than the classic proline rich motif PxxP [23]. The C-terminal “effector region” of EPS8 can facilitate the Rac-activating complex SOS1/EPS8/ABI1 to its proper subcellular site (actin filaments), and affect the substrate specificity of SOS1 [22]. ABI1 was reported to be a general coordinator of kinase-substrate interactions. It is involved in the formation of many macromolecule complexes related to cytoskeleton regulation, such as Abl/ABI1/WAVE2 [39], Napl/PIRl21/ABI1/WAVE [40], and c-Abl/ABI1/Mena [41]. The N-terminal of ABI1 consists of WAB (wave binding domain), SNARE, and HHR domains. Moreover, there is a non-classic proline rich motif PxxDY located in the C-terminal of ABI1, which can specifically bind to the SH3 domain of EPS8 [26]. The C-terminal of ABI also has a poly-proline structure and a SH3 site, which can bind to carboxy-terminal portion of Abl [47].

Although Scita et al. had suggested that the SOS1/ABI1/EPS8 complex mediated the signal transduction from Ras to Rac in 1999 [20], the involved molecular mechanism is not well understood. They thought that ABI1 bond to the proline rich domain of SOS1 through its SH3 domain and to the SH3 domain of EPS8 through its proline domain. They also suggested that ABI1 might serve as a scaffold protein connecting SOS1 and EPS8 [22]. This model was verified by Innocenti et al. later [17, 18], but various questions remained to be answered. First, the existence of the tri-complex under physiological conditions should be verified. In this study, we performed Co-IP in metastatic ovarian cancer cell line SK-OV3 after LPA stimulation. The results showed that both SOS1 and EPS8 were detected in the anti-ABI1 immunoprecipitates. Then, we further demonstrated that there were interactions between ABI1-SOS1 and ABI1-EPS8, but SOS1 and EPS8 could not directly bind with each other. These results proved the existence of an endogenous SOS1/EPS8/ABI1 tri-complex, and the role of ABI1 as a scaffold protein holding together SOS1 and EPS8. To further investigate the binding sites in ABI1 for SOS1 and EPS8, we divided ABI1 into different regions, and used Co-IP assay to detect the regions responsible for protein interactions. Our results indicated that the SH3 region of ABI1 mediated ABI1-SOS1 binding, as described by the previous studies. We also found that the poly-proline+PxxDY region of ABI1 was responsible for its interaction with EPS8, which had not been reported yet. Besides that, we also demonstrated that the proline-rich region of SOS1 and the SH3 region of EPS8 mediated their interactions with ABI1, respectively.

In the recent years, inhibitory peptides have been successfully used to specifically disrupt signaling complexes, thereby blocking relevant biological events, which provides valuable insights into the development of peptide-type drugs [33, 34]. Various cargo sequences including the HIV-TAT sequence have been employed to assist the inhibitory peptides in penetrating the plasma membrane [45]. Since none of members in SOS1/EPS8/ABI1 tri-complex is dispensable in the Rac activation, and ABI1 acts as a scaffold protein, we hypothesize that ABI1 may be an ideal target to design anti-tumor drugs.

Based on the regions of ABI1 that were found responsible for SOS1 and EPS8 bindings, we generated a series of synthetic inhibitory short peptides. Efficient inhibitory short peptides were supposed to disrupt the protein-protein interactions. Through GST-pulldown assay and Co-IP, the short peptide p + p-8 (ppppppppvdyedee) was identified as the one capable of blocking ABI1-EPS8 interaction, while SH3–3 (ekvvaiydytkdkddelsfmegaii) as the one could effectively inhibit the combination of ABI1 and SOS1 in vitro.

Subsequently, we used a HIV-TAT sequence (YGKKRRQRRPP), an effective cell membrane penetrating peptide, to modify the screened inhibitory short peptides, so as to transfer the peptides into ovarian cancer cells. The results showed that the peptide TAT-p + p-8, which was capable of disrupting ABI1-EPS8 interaction in vitro, could also efficiently block the binding of ABI1-EPS8 in vivo. However, the peptide TAT-SH3–3 was not efficiently enough to block the interaction between ABI1 and SOS1 in vivo. The longer length and weaker penetration capability of the peptide might be the reason for the worse inhibitory efficiency of the peptide.

Since the purpose of our study was to eventually block the downstream signaling pathways and biological events mediated by the SOS1/EPS8/ABI1 tri-complex through disrupting proteins interactions, we evaluated the efficacy of the selected inhibitory short peptides in suppressing ovarian cancer metastasis. Using Matrigel invasion assay as well as peritoneal metastatic colonization model, we found that the inhibitory short peptide TAT-p + p-8 could effectively suppress the invasion and metastasis of ovarian cancer cells. However, the inhibitory effects of peptide TAT-SH3–3 on cell invasion and metastatic colonization was not as statistically significant as TAT-p + p-8. Since peptide can be degradation by enzymes, and the stability of peptides mainly depends on primary and secondary structure. Thus, peptide SH-3 may be more easily degraded by enzymes, resulting in poor inhibitory potential in vivo. In the following studies, we will adopt chemical strategies, such as N and C termini modifications, to modify the peptides to increase their in vivo stabilities. Then the stability and pharmacokinetic profile of the peptide will be evaluated.

## Conclusions

The specific involvement of EPS8/ABI1/SOS1 tri-complex in ovarian cancer metastasis suggests that this tri-complex-targeted drugs may have less effects on non-cancer tissues than on cancer tissues. As an antagonist of the parental protein for the same binding site, short peptides have high specificity and affinity to the targeting proteins. This is the basis on which short peptides are expected to serve as a starting point for pharmacochemistry. However, short peptides may still have potential toxicity to normal tissues, which needs to be further evaluated in future studies. The fact that ovarian cancer is mainly confined to the peritoneal cavity [5, 6] ensures the feasibility of locally delivering therapeutic peptides at effective dosages. This study may provide a theoretical basis for the future development of anticancer drugs.

## Data Availability

The data supporting the conclusions of this article is included within the article and supplementary material.

## Abbreviations

DMEM: Dulbecco’s modified Eagle’s medium
FBS: Fetal bovine serum
FBSL: Fetal bovine serum
GEF: Guanosine monophosphate exchange factor
IPTG: Isopropy-β-D-thiogalactoside
LPA: Lysophosphatic acid
OC: Ovarian cancer
